# Quality of life indicators among recent Ghanaian stroke survivors across 10 hospitals: A cross-sectional study^✩^

**DOI:** 10.1016/j.neuros.2026.100026

**Published:** 2026-03-26

**Authors:** Nana Kwame Ayisi-Boateng, Douglas Aninng Opoku, Jude Domfeh Darkwah, Albert Akpalu, Ansumana Bockarie, Emmanuel Konadu, Rexford Adu-Gyamfi, Agnes Arthur, Kwadwo Agyenim-Boateng, Christiana Neizer, Timothy Fiattor, Priscilla Opare-Addo, Nyantakyi Adu-Darko, Nathaniel Adusei-Mensah, Raelle Tagge, Michael Ampofo, Hilda Kwapong, John Akassi, John Humphrey Amuasi, Samuel Blay Nguah, Bruce Ovbiagele, Fred Stephen Sarfo

**Affiliations:** a Kwame Nkrumah University of Science and Technology, Kumasi, Ghana; b Kwame Nkrumah University of Science & Technology Hospital, Kumasi, Ghana; c University of Ghana, Accra, Ghana; d University of Cape Coast, Cape Coast, Ghana; e Agogo Presbyterian Hospital, Agogo, Ghana; f Ankaase Methodist Faith Hospital, Ankaase, Ghana; g Kwadaso SDA Hospital, Kumasi, Ghana; h Kumasi South Hospital, Kumasi, Ghana; i Manhyia District Hospital, Kumasi, Ghana; j Komfo Anokye Teaching Hospital, Kumasi, Ghana; k Northern California Institute of Research and Education, USA; l Kumasi Centre for Collaborative Research, Kumasi, Ghana; m University of California, San Francisco, USA

**Keywords:** EQ-5D, EQ-VAS, Ghana, Quality of life, Recent stroke survivors

## Abstract

**Background:**

Despite the increased burden of stroke in sub-Saharan Africa, studies assessing patient-centered outcomes such as quality of life (QoL) in the population are limited. We sought to assess the QoL, compare domain scores by sex, as well as their determinants among recent stroke survivors in Ghana.

**Methods:**

This multi-centre cross-sectional study was conducted in 10 health facilities in Ghana. We used the European Quality of Life Scale-5 Dimensions (EQ-5D) to assess the QoL among 500 individuals who had survived a stroke within one month of symptom onset and had uncontrolled hypertension. Factors significantly associated with QoL were assessed using multivariable linear regression.

**Results:**

The mean age of males was 58.0 (±11.0) vs 59.0 (±12.0) years among the females. Usual activity (1.9 ± 0.7) and mobility (1.8 ± 0.7) had the highest mean scores for perceived problems, whereas anxiety/depression (1.4 ± 0.6) had the lowest. Over half (56.1%) of the stroke survivors rated their EQ-VAS score at or above the median. After adjusting for significant covariates in the multivariate linear regression analysis, female sex (*β*: 8.90, 95%CI: 4.30, 14.00), living in urban areas (*β*: 13.00, 95%CI: 5.10, 21.00), having a monthly income of 251 – 500 USD (*β*: 7.50, 95%CI: 1.80, 13.00) and having higher Barthel index (*β*: 0.21, 95%CI: 0.13, 0.29) were significantly associated with better QoL (EQ-VAS score). In sex-stratified analyses, male participants living in semi-urban or urban areas, with higher income, and with a higher Barthel index were significantly associated with better QoL. Among female participants, having an untyped stroke type was associated with lower QoL, whereas a higher Barthel index was associated with better QoL.

**Conclusion:**

Functional independence, sociodemographic factors, and sex affect the QoL of this cohort of recent stroke survivors with uncontrolled hypertension in Ghana. Living in urban areas, higher functional independence, female sex, and higher income were associated with better QoL. In contrast, having an untyped CT scan was associated with lower QoL among women. Socioeconomic factors and prior health problems in recent stroke survivors significantly influence QoL. Post-stroke QoL may be enhanced by strategies that improve functional recovery and address the social determinants of health.

## Background

Stroke is one of the major causes of disability and premature deaths, with an annual global mortality of 6.55 million [1]. It poses a public health concern due to its severe impacts on survivors’ functional independence and quality of life (QoL) [2–4]. Additionally, stroke survivors have an increased risk of functional impairment, depression, and social isolation [5–7].

Aside from optimizing the functional independence of stroke survivors, enhancing their QoL has gained attention in research and clinical practice in terms of the management and recovery process of stroke survivors [2,8]. QoL assessment provides details on patients’ health status across physical, functional, cognitive, social, psychological well-being, and environmental domains, and assesses the impact of treatment modalities using the individual’s perceptions [9,10]. Various studies measuring the QoL of stroke patients have reported varied results, which could be due to variations in healthcare systems, socio-economic, and cultural factors [2,11–13]. Earlier studies on the QoL of stroke patients have reported the psychological [14], mobility [2] and physical domains [13,15] as the most significant problem areas requiring attention in their management and rehabilitation. There is overwhelming evidence to suggest that an interplay of several factors influences the QoL of stroke survivors. Factors such as older age, depression, stroke severity, recurrent stroke, low level of education, and poor socio-economic status have been reported to be associated with poor QoL among stroke survivors [2,11,16–18]. Multiple studies conducted in high-income settings have shown consistently lower QoL among female stroke survivors compared with males after adjusting for important socio-economic and stroke severity predictors [19–29].

Despite studies reporting an increased burden of stroke in Ghana [30–32], there is limited data on studies measuring the QoL of individuals who survived the event. Among the few studies investigating QoL among Ghanaian stroke survivors, Donkor et al. reported higher health-related QoL scores across all domains among healthy patients than among stroke survivors in two public health facilities in the Greater Accra Region of Ghana [33]. Akpalu et al. also reported a significant association between cognitive impairment and poor QoL among Ghanaian stroke survivors in the Korle Bu Teaching Hospital [34]. These studies, however, did not estimate the functional independence and its effect on the QoL of stroke survivors, and the known disparities in QoL by sex differences were not explored [33,34]. Further studies to estimate QoL and associated factors are imperative to enrich the literature, especially from a resource-limited setting perspective, and to guide clinicians in making management and rehabilitation decisions for stroke survivors. Hence, our study aimed to: (1) assess the QoL of recent stroke survivors in Ghana; (2) identify factors associated with QoL, including functional independence; and (3) examine sex-specific differences in QoL across 10 hospitals representing the various cadres of healthcare delivery in the country.

## Methods

### Study design

This study utilized a cross-sectional design to assess the QoL of recent stroke survivors using baseline data from a Phase III Randomized Controlled Trial – Phone-Based Intervention Under Nurse Guidance After Stroke (PINGS II) [35].

### Study setting

The study was conducted at 10 sites, representing the three levels of healthcare delivery in Ghana: tertiary (3), secondary (2), and primary level (5) facilities located in urban, peri-urban, and rural communities. The tertiary hospitals were the Korle Bu Teaching Hospital, Komfo Anokye Teaching Hospital, and Cape Coast Teaching Hospital. The secondary level hospitals were Agogo Presbyterian Hospital and Kumasi South Hospital. The primary-level facilities were Methodist Hospital, Ankaase Methodist Faith Hospital, Manhyia District Hospital, the University Hospital, Kwame Nkrumah University of Science and Technology (KNUST), Kwadaso Seventh-Day Adventist Hospital, and Tafo Government Hospital.

### Study population and sample size

The study participants were 18 years and above, within one month of stroke onset, and had uncontrolled hypertension (blood pressure ≥ 140/90 mmHg). They all had ischemic or hemorrhagic strokes based on brain imaging. In a few cases where diagnostic imaging was not available, stroke was diagnosed clinically using the locally validated version of the 8-item questionnaire for verifying stroke-free status (8-QVSFS) [36]. Stroke survivors with severe cognitive impairment/dementia, including those who could not meaningfully engage due to diminished alertness, were excluded from the trial. Cognitive function was assessed using the Modified Mini-Mental State Examination (MMSE), with a score of <24 used as the cut-off for severe cognitive impairment. We included patients with aphasia who did not have severe cognitive impairment, and, where necessary, engaged caregivers to facilitate efficient communication and reliable data collection.

### Sample size and participant recruitment

Our sample size was based on the published study protocol for the main study (24). We obtained data from 500 participants enrolled in the Phase III randomized controlled trial, Phone-Based Intervention Under Nurse Guidance After Stroke (PINGS II) (24). Study participants were recruited from all 10 sites in accordance with the inclusion and exclusion criteria. They were recruited on an outpatient basis within one month of suffering a stroke and discharged from the ward. Specifically, participants’ recruitment for the baseline data commenced on October 23, 2020, and concluded on December 4, 2023.

### Data collection

Data on participants’ sociodemographic characteristics, including age, sex, education level, marital status, and clinical data such as stroke type and location, were collected using a Case Record Form. The European Quality of Life Scale-5 Dimensions (EQ-5D) was used to assess participants’ QoL [37]. This is a validated and widely used tool to assess the current health status (QoL) of stroke survivors across the globe [2,38,39]. This tool comprises two components: the EQ-5D descriptive system and the EQ Visual Analogue Scale (EQ-VAS). The EQ-5D assesses one’s current health in five domains: mobility, self-care, usual activity (e.g., work, study, housework, family or leisure), pain/discomfort, and anxiety/depression [37]. The stroke survivors were asked to rate their current health using a five-point Likert scale, with a higher rating indicating the severity of the problem, ranging from 1 (no problem) to 5 (severe problem) [2]. The stroke survivors were asked to rate their overall health today using the EQ-VAS, which ranges from 0 (worst) to 100 (best). The Barthel Index was also used to assess functional independence in stroke survivors. This tool measures activities of daily living or functional independence in the domains of mobility and personal care of patients with chronic conditions such as stroke [40,41]. The Barthel Index is a ten-item scale with a minimum score of 0 and a maximum score of 100, with a higher score indicating more functional independence [40,41].

### Analysis

Data were entered into REDCap, and data were double-checked to enhance data quality. Data analysis was performed using RStudio version R.3.6.0. Categorical data were summarized as frequencies and percentages, whereas continuous data were summarized as medians, means, and standard deviations. Chi-square tests, Fisher’s exact tests, and Wilcoxon rank-sum tests were used to compare sociodemographic and clinical characteristics, EQ-5D, VAS, and the Barthel Index between male and female participants, as appropriate. In this study, EQ-VAS scores were categorized as below or at or above the study sample median. Scores at the median or above indicate a better QoL, whereas scores below reflect a poorer QoL. Univariate and multivariate linear regression analyses were used to identify factors significantly associated with participants’ QoL. First, a univariate linear regression analysis was performed to identify significant covariates. All significant covariates were included in the multivariable linear regression model and adjusted for. We considered covariates with p-values < 0.05 as statistically significant in all analyses.

### Ethical approval

This study was granted ethical approval by the Committee on Human Research Publications and Ethics (CHRPE) of the School of Medical Sciences, Kwame Nkrumah University of Science and Technology, the Ethics Review Committee of the Ghana Health Service, and the Institutional Review Boards of the Komfo Anokye Teaching Hospital, Cape Coast Teaching Hospital, and the Korle Bu Teaching Hospital. All study participants provided informed consent by signing or thumb-printing a consent form before being recruited.

## Results

### Socio-demographic and clinical characteristics of study participants

Among the 500 stroke survivors recruited for the study, the mean age of males was 58.0 (±11.0) years, compared with 59.0 (±12.0) years among females. The proportion of married study participants was higher among males than among females (83.6 % vs. 44.7 %), while those with primary education were higher among females than among males (52.1 % vs. 32.0 %). Approximately 70.8 % of males lived with their spouses and children, compared with 33.8 % of females. Regarding the stroke type, ischemic stroke was higher in females (82.8 %) than in males (67.4 %). Regarding stroke location, the majority of males (71.0 %) and females (80.1 %) had their strokes in the anterior circulation. (Table 1).

### Quality of life and activities of daily living among the study participants

Among the domains of the EQ-5D for the levels of perceived problems, usual activity (1.9 ± 0.7) and mobility (1.9 ± 0.7) were the highest-rated mean scores, while anxiety/depression (1.4 ± 0.6) was the lowest-rated mean score. For males and females, the mean mobility score was (1.7 ± 0.7) and (1.8 ± 0.7), respectively. The mean self-care scores for males and females were 1.7 ± 0.7 and 1.7 ± 0.8, respectively. Over half (56.1 %) of the stroke survivors rated their EQ-VAS score median and above, with both males and females rating their EQ-VAS score median and above. The mean Barthel index was 66.0 ± 27.0 (Table 2).

### Determinants of quality of life among the study participants

In the univariate linear regression analysis, participants’ age, gender, domicile, income, stroke type, and Barthel index were associated with QoL. In the multivariate linear regression analysis, factors such female sex (*β*: 8.90, 95 %CI: 4.30, 14.00), living in urban areas (*β*: 13.00, 95 %CI: 5.10, 21.00), monthly income of 251 – 500 USD (*β*: 7.50, 95 %CI: 1.80, 13.00) and Barthel index (*β*: 0.21, 95 %CI: 0.13, 0.29) were significantly associated with better QoL (Table 3).

Multivariate linear regression modelling in males showed that factors such as living in semi-urban (*β*: 20.00, 95 %CI: 8.20, 32.00) and urban (*β*: 18.00, 95 %CI: 6.40, 29.00) areas, 101 – 250 USD (*β*: 8.20, 95 %CI: 1.20, 15.00) and 251 – 500 USD (*β*: 10.00, 95 %CI: 2.40, 18.00) monthly income levels, and Barthel index (*β*: 0.21, 95 %CI: 0.09, 0.32) were significantly associated with better QoL while in females, having an untyped Stroke (*β*: −29.00, 95 %CI: 58.00, −0.66) was associated with worse QOL. In contrast, female participants with a higher Barthel index (*β*: 0.22, 95 %CI: 0.12, 0.33) were independently associated with better QoL (Table 4a, Table 4b).

## Discussion

Overall, the QoL of the study participants was good, with over half (56.1 %) rating their overall health as median or above, based on the VAS scores. Our study revealed that the recent stroke survivors in this population experienced challenges across several domains of QoL, with the highest and lowest mean scores reported in the usual activity and anxiety/depression domains, respectively. It is plausible that the early timing of the assessment (within one month of stroke onset) may have contributed to the relatively lower rate of anxiety/depression observed among the study participants, as mood disorders may be more noticeable while in the subacute to chronic phases. Additionally, sociocultural determinants related to how mood disorders are perceived and reported in the Ghanaian context may have contributed to these findings. Importantly, participants were drawn from all three levels of healthcare delivery in Ghana: primary, secondary, and tertiary. Differences in the quality of care received across these levels may have influenced outcomes and perceived QoL. After usual activity, mobility was the next most prevalent health problem in this population, consistent with findings among stroke survivors in Malaysia and Australia, where mobility and usual activity were the most rated across the EQ-5D dimensions [2,15]. Mobility and usual activity being the most reported health problem of the five domains in our study is not surprising, given that the study population was recent stroke survivors, with most having complaints of numbness, weakness, or paralysis in the upper or lower limb. The mobility and usual activity domains include performing daily or routine activities, such as work, cooking, and leisure, which require the ability to walk or move. Notably, a previous study in Bangladesh among recent stroke patients reported that 79.5 % and 87.0 % of them had health problems with mobility and usual activity, respectively [39].

Our study also examined sex differences in QoL, VAS, and the Barthel Index among study participants. Among the EQ-5D dimensions, only the pain/discomfort domain showed a statistically significant sex difference, with women reporting higher levels than men. This finding suggests that women may report or suffer more pain/discomfort regularly than men, indicating a likely unmet necessity for enhanced pain care among the female patients in this study. Our finding corroborates with studies that have demonstrated that female stroke survivors often report higher pain/discomfort than males [42,43]. Factors such as biological and hormonal differences, psychological factors, and health-seeking behaviours may have accounted for the observed sex difference in this study. Female stroke patients may benefit from sex-sensitive methods of pain assessment and care.

In our study, participants earning 251–500 USD per month had QoL scores that were 8 times higher than those earning ≤100 USD. This finding underscores earlier reports that income level is a significant determinant of QoL among stroke survivors [44–46]. A possible explanation for this finding is that individuals with higher monthly incomes (251–500 USD) may have greater purchasing power than those earning ≤100 USD, increasing their ability to access quality healthcare and other facilities that enhance their QoL. Although the National Health Insurance Scheme in Ghana was established to improve access to healthcare, gaps in coverage persist, and some insured patients still incur out-of-pocket expenses for services such as rehabilitation, diagnostics, and medications [47]. Especially, many rehabilitation services, except physiotherapy, are excluded under the National Health Insurance Scheme, and rehabilitation infrastructure and professionals are more readily available in tertiary facilities in urban centres [48,49], creating limited access for those in rural areas and lower-income groups.

Functional independence plays a pivotal role in assessing the overall health of stroke survivors. In this study, we found that a unit increase in the Barthel index score was associated with higher QoL scores. This suggests that functional independence among recent stroke survivors in our study was positively associated with QoL. This corroborates findings from studies conducted in Germany and Turkey that demonstrated that functional independence is a predictor of QoL among stroke survivors [50,51].

In this study, compared to those living in rural areas, those who were residing in either urban or semi-urban areas had their QoL scores increased by five and three times, respectively, comparable to studies conducted elsewhere [52,53]. However, a sex-stratified analysis revealed that this association was significant only among men, suggesting that men may benefit more from living in semi-urban or urban areas with respect to QoL. Residing in urban or semi-urban areas may be associated with higher socioeconomic status and employment, which could influence one’s ability to access quality healthcare, including purchasing high-cost drugs/treatment [54,55]. These findings underscore the need for improved equitable access to healthcare in Ghana. The observed significant association among only males in this study suggests that sex and place of residence may interact to influence post-stroke outcomes, such as QoL. The socio-economic advantages of semi-urban or urban settings, such as job prospects, mobility, and enhanced accessibility to healthcare, may disproportionately favour men.

The sex-stratified analysis further revealed that among females, those who had an untyped stroke had poorer QoL compared to those diagnosed with ischemic stroke. This finding may be due to health-system and care-pathway barriers rather than to stroke type alone. In resource-constrained settings, inadequate access to neuroimaging is often associated with late presentation, advanced stroke severity, and rehabilitation, all of which have been reported to affect stroke outcomes such as QoL [56–58]. These challenges may be particularly pronounced for females who have additional constraints to healthcare accessibility, including financial barriers and caregiving duties [59–61].

Interestingly, unlike previous studies reporting higher QoL in males than in females [62,63]. Our study found that female sex was associated with higher QoL scores than male sex. This can be attributed to sociocultural differences in the roles and responsibilities that women play across societies. For instance, in Ghanaian culture, males in the age group of our study population are more likely to be the primary breadwinners for their families. They may perceive the stroke as more catastrophic, resulting in deterioration of their QoL. However, our results compare favourably with studies conducted in Denmark and Sweden, where being a female stroke survivor was associated with better QoL [64,65]. Further research is needed to gain a more nuanced understanding of how socio-cultural factors influence sex differences in post-stroke QoL.

Additionally, our results revealed notable disparities in Ghanaian post-stroke QoL, especially across sex, marital status, place of residence, and socio-economic status. Despite EQ-5D being a widely validated tool, it may not completely capture culturally specific dimensions of QoL, including community involvement and family support systems, which are particularly significant in the sub-Saharan context. Prioritizing the development and use of sex-sensitive and culturally appropriate tools for assessing early-onset post-stroke outcomes may enhance the accuracy and applicability of patient-centered outcome measures in this setting.

### Strengths and limitations of the study

One limitation of this study is that, given its cross-sectional design, we cannot establish a cause-and-effect relationship. The exclusion of stroke survivors with cognitive impairment (MMSE <24) led to the recruitment of those who had a relatively better cognitive function, which may have biased the observed QoL outcome. Additionally, the assessment of QoL using the EQ-5D and EQ-VAS is a self-reported measure and thus subject to response bias. Assessment of QoL only during the early post-stroke period may have influenced the estimates. However, a strength of this study is that participants were enrolled from primary, secondary, and tertiary levels of care across the Ghanaian health system, thereby enhancing the generalizability of our findings to this defined population. Another strength of this study is the sex-stratified analyses, which provide deeper insights into the gender-based differences in QoL, which is insufficiently studied in the Ghanaian setting among recent stroke survivors. Our findings lay a foundation for developing more targeted policies or strategies for stroke care in Ghana and beyond. Future studies can employ a longitudinal design to examine how promoting physical support among recent stroke survivors affects their QoL over time. Although clinical trials among stroke survivors in this region are sparse, investigators should consider including measures of QoL as a patient-centered outcome measure.

## Conclusion

Quality of life is negatively affected even in the early period following stroke onset. Among our study population, the usual activity and mobility domains were the most prevalent health problems. Additionally, female sex, living in urban areas, the Barthel Index, and monthly income level were significantly associated with QoL among the stroke survivors. Sex-specific analyses also revealed that, among male participants, living in semi-urban or urban areas, monthly income, and the Barthel Index were significantly associated with QoL. In contrast, among females, an untyped CT scan was associated with lower QoL, whereas the Barthel Index was independently associated with QoL. We recommend promoting physical, emotional, and financial support from family members or caregivers for recent stroke survivors. Additionally, we recommend improving rehabilitation services in rural areas, given the observed disparities in participants’ residence between urban and rural areas in our study. We also recommend implementing sex-specific post-stroke interventions that address the unique social and economic challenges experienced by both men and women to improve their QoL.

## Figures and Tables

**Table 1:** Socio-demographic and clinical characteristics of the study participants.

Characteristic	Sex of study participant		Overall, *N* = 500	p-value^2^
	Male, *N* = 281^1^	Female, *N* = 219^1^		

**Age in years**	58 (11.0)	59(12.0)	58 (12)	0.094
**Marital Status**				**<0.001**
Currently Married	235 (83.6)	98 (44.7)	333 (66.6)	
Previously Married	32 (11.4)	112 (51.1)	144 (28.8)	
Never Married	14 (5.0)	9 (4.1)	23 (4.6)	
**Educational Status**				**<0.001**
None	11 (3.9)	38 (17.4)	49 (9.8)	
Primary	90 (32.0)	114 (52.1)	204 (40.8)	
Secondary	113 (40.2)	51 (23.3)	164 (32.8)	
Tertiary	67 (23.8)	16 (7.3)	83 (16.6)	
**Living Status**				**<0.001**
Lives Alone	16 (5.7)	13 (5.9)	29 (5.8)	
Lives With Spouse and Children	199 (70.8)	74 (33.8)	273 (54.6)	
Lives With Spouse only	18 (6.4)	12 (5.5)	30 (6.0)	
Lives With Extended Family	30 (10.7)	42 (19.2)	72 (14.4)	
Lives With Children only	18 (6.4)	78 (35.6)	96 (19.2)	
**Religion**				0.901
Christianity	250 (89.0)	198 (90.4)	448 (89.6)	
Islam	29 (10.3)	20 (9.1)	49 (9.8)	
Other	2 (0.7)	1 (0.5)	3 (0.6)	
**Domicile**				0.421
Rural	16 (5.7)	17 (7.8)	33 (6.6)	
Semi-Urban	99 (35.2)	67 (30.6)	166 (33.2)	
Urban	166 (59.1)	135 (61.6)	301 (60.2)	
**Monthly Income in USD**				**0.028**
0–100	83 (29.6)	91 (42.1)	174 (35.1)	
101–250	88 (31.4)	61 (28.2)	149 (30.0)	
251–500	67 (23.9)	42 (19.4)	109 (22.0)	
>500	42 (15.0)	22 (10.2)	64 (12.9)	
**Stroke Type (*n* = 434)**				**0.006**
Ischemic Stroke	167 (67.4)	164 (82.8)	342 (76.0)	
Intracerebral Hemorrhagic Stroke	71 (28.2)	32 (16.2)	103 (22.9)	
Untyped Stroke	3 (1.2)	2 (1.0)	5 (1.1)	
**Stroke Location (*n* = 368)**				**0.045**
Anterior Circulation	147 (71.0)	129 (80.1)	276 (75.0)	
Posterior Circulation	60 (29.0)	32 (19.9)	92 (25.0)	
**Body Mass Index**	25.5 (4.8)	28.0 (6.1)	26.6 (5.5)	**<0.001**
**NIHSS (mean ± SD)**	3.0 (0.0, 8.0)	3.0 (0.0, 7.5)	3.0 (0.0, 8.0)	0.757

1 Mean (SD); n ( %); Median (Q1, Q3); NIHSS: National Institute of Health Stroke scale.

2 Wilcoxon rank sum test; Pearson’s Chi-squared test; Fisher’s exact testUntyped stroke: Stroke without computed tomography (CT) scan confirmation.

**Table 2:** Descriptive statistics of EQ-5D, VAS and Barthel index scales.

Characteristic	Sex of study participant		Overall, *N* = 500^1^	p-value^2^
	Male, *N* = 281^1^	Female, *N* = 219^1^		

**Mobility**	1.7 (0.7)	1.8 (0.7)	1.8 (0.7)	0.131
**Self-Care**	1.7 (0.7)	1.7 (0.8)	1.7 (0.7)	0.734
**Usual Activity**	1.9 (0.7)	1.9 (0.7)	1.9 (0.7)	0.306
**Pain Discomfort**	1.6 (0.6)	1.7 (0.6)	1.6 (0.6)	**0.024**
**Anxiety/Depression**	1.4 (0.6)	1.4 (0.6)	1.4 (0.6)	0.787
**Your Health Today**	64.0 (23.0)	68.0 (22.0)	65.0 (23.0)	**0.040**
**Categorized VAS**				0.076^2^
Below Median	133.0 (47.5)	85.0 (39.2)	218.0 (43.9)	
Median & above	147.0 (52.5)	132.0 (60.8)	279.0 (56.1)	
**Barthels Index**	68.0 (26.0)	65.0 (29.0)	66.0 (27.0)	0.458

1 Mean (SD); n ( %).

2 Wilcoxon rank sum test; Pearson’s Chi-squared test.

**Table 3:** Univariate and multivariate linear regression analysis of factors associated with quality of life of study participants.

Variables	N	Univariate			Multivariate		
		Estimate	95 % CI^1^	p-value	Beta	95 % CI^1^	p-value

**Age in years**	497	−0.27	−0.44, −0.10	**0.002**	−0.17	−0.35, 0.02	0.074
**Gender**	497						
Male		1.00			1.00		
Female		3.90	−0.11, 7.90	0.056	8.90	4.30, 14.00	**< 0.001**
**Marital Status**	497						
Currently Married		1.00					
Previously Married		−2.50	−7.00, 2.00	0.264			
Never Married		−5.30	−15.00, 4.30	0.279			
**Educational Status**	497						
None		1.00					
Primary		3.60	−3.60, 11.00	0.324			
Secondary		5.20	−2.20, 13.00	0.173			
Tertiary		4.80	−3.30, 13.00	0.244			
**Living Status**	497						
Lives Alone		1.00			1.00	1.00	
Lives With Spouse and Children		−5.80	−14.00, 2.90	0.191	0.04	−8.90, 9.00	0.993
Lives With Spouse		−11.00	−23.00, 0.17	0.053	−4.50	−16.00, 7.00	0.443
Lives With Extended Family		−8.20	−18.00, 1.60	0.097	−3.60	−14.00, 6.50	0.484
Lives With Children		−11.00	−20.00, −1.40	**0.024**	−7.30	−17.00, 2.60	0.148
**Religion**	497						
Christianity		1.00					
Islam		−4.60	−11.00, 2.10	0.179			
Other		−2.60	−28.00, 23.00	0.846			
**Domicile**	497						
Rural		1.00			1.00		
Semi-Urban		11.00	2.40, 19.00	**0.012**	11.00	3.00, 19.00	**0.007**
Urban		12.00	3.40, 20.00	**0.006**	13.00	5.10, 21.00	**0.001**
**Monthly Income in USD**	493						
0–100		1.00			1.00	—	
101–250		10.00	5.20, 15.00	**<0.001**	6.40	1.30, 11.00	**0.014**
251–500		9.80	4.50, 15.00	**<0.001**	7.50	1.80, 13.00	**0.010**
>500		1.40	−5.00, 7.9.00	0.661	0.21	−7.00, 7.40	0.955
**Stroke Type**	432						
Ischemic Stroke		1.00			1.00		
Intracerebral Hemorrhagic Stroke		4.50	−0.45, 9.50	0.074	−0.51	−5.6, 4.5	0.842
−23–43, −3.2**0.023**–18-36, 0.280.054Untyped Stroke		−23.00	−43.00, −3.20	**0.023**	−18.00	−36.00, 0.28	0.054
**Body Mass Index**	478	0.34	−0.03, 0.71	0.074	0.05	−0.33, 0.44	0.778
**Barthels Index**	482	0.25	0.18, 0.32	**<0.001**	0.21	0.13, 0.29	**<0.001**

1 CI = Confidence Interval.

**Table 4a:** Univariate and multivariate linear regression analysis of factors associated with quality of life of male study participants.

Variables	N	Univariate			Multivariate		
		Estimate	95 % CI^1^	p-value	Beta	95 % CI^1^	p-value

**Age in years**	280	−0.30	−0.53, −0.06	**0.014**	−0.15	−0.41, 0.10	0.229
**Marital Status**	280						
Currently Married		1.00					
Previously Married		−0.12	−8.70, 8.50	0.950			
Never Married		−8.80	−21.00, 3.70	0.166			
**Educational Status**	280			0.688			
None		1.00					
Primary		−0.56	−15.00, 14.00	0.942			
Secondary		3.20	−11.00, 18.00	0.668			
Tertiary		2.50	−12.00, 17.00	0.742			
**Living Status**	280						
Lives Alone		1.00			1.00		
Lives With Spouse and Children		−7.70	−19.00, 4.10	0.200	1.10	−11.00, 13.00	0.849
Lives With Spouse		−16.00	−32.00, −0.86	**0.039**	−5.60	−21.00, 9.80	0.475
Lives With Extended Family		−9.2.0	−23.00, 4.90	0.191	6.50	−7.60, 21.00	0.364
Lives With Children		−16.00	−31.00, −0.13	**0.048**	−3.10	−19.00, 12.00	0.695
**Religion**	280						
Christianity		1.00					
Islam		−3.10	−12.00, 5.90	0.504			
Other		6.00	−26.00, 38.00	0.714			
**Domicile**	280						
Rural		1.00			1.00		
Semi-Urban		20.00	8.20, 32.00	**0.001**	20.00	8.20, 32.00	**<0.001**
Urban		16.00	3.80, 27.00	**0.010**	18.00	6.40, 29.00	**0.002**
**Monthly Income in USD**	279						
0–100		1.00			1.00	1.00	
101–250		11.00	4.20, 18.00	**0.002**	8.20	1.20, 15.00	**0.021**
251–500		13.00	5.40, 20.00	**<0.001**	10.00	2.40, 18.00	**0.011**
> 500		7.6.00	−0.94, 16.00	0.080	4.30	−5.00, 14.00	0.361
**Stroke Type**	245						
Ischemic Stroke		1.00					
Intracerebral Hemorrhagic Stroke		5.80	−0.56, 12.00	0.074	1.20	−5.30, 7.60	0.725
Untyped Stroke		−17.00	−43.00, 9.50	0.209	−8.80	−34.00, 16.00	0.491
**Body Mass Index**	273	−0.23	−0.81, 0.35	0.433			
**Barthels Index**	268	0.28	0.18, 0.38	**<0.001**	0.21	0.09, 0.32	**<0.001**

1 CI = Confidence Interval.

**Table 4b:** Univariate and multivariate linear regression of factors associated with quality of life of female study participants.

Variables	N	Univariate			Multivariate		
		Estimate	95 % CI^1^	p-value	Beta	95 % CI^1^	p-value

**Age in years**	217	−0.26	−0.52, −0.01	**0.040**	−0.01	−0.30, 0.28	0.945
**Marital Status**	217						
Currently Married		1.00			1.00		
Previously Married		−7.90	−14.00, −1.90	**0.010**	−3.40	−14.00, 7.30	0.532
Never Married		−1.80	−17.00, 13.00	0.817	3.70	−14.00, 21.00	0.683
**Educational Status**	217						
None		1.00			1.00		
Primary		6.60	−1.60, 15.00	0.115	7.50	−0.80, 16.00	0.076
Secondary		8.40	−1.00, 18.00	0.080	7.20	−2.40, 17.00	0.139
Tertiary		12.00	−0.55, 25.00	0.060	10.00	−2.80, 23.00	0.125
**Living Status**	217						
Lives Alone		1.00			1.00		
Lives With Spouse and Children		−0.18	−13.00, 13.00	0.978	−3.80	−20.00, 13.00	0.648
Lives With Spouse		−3.80	−21.00, 13.00	0.662	−7.00	−26.00, 12.00	0.479
Lives With Extended Family		−7.70	−21.00, 6.00	0.268	−12.00	−27.00, 2.00	0.092
Lives With Children		−10.00	−23.00, 2.80	0.125	−12.00	−25.00, 1.20	0.075
**Religion**	217						
Christianity		1.00					
Islam		−6.50	−17.00, 3.70	0.210			
Other		−18.00	−62.00, 25.00	0.409			
**Domicile**	217						
Rural		1.00			1.00		
Semi-Urban		1.10	−11.00, 13.00	0.859	−0.85	−12.00, 10.00	0.882
Urban		8.80	−2.30, 20.00	0.119	5.80	−4.90, 16.00	0.285
**Monthly Income in USD**	214						
0–100		1.00					
101–250		11.00	4.00, 18.00	**0.002**	3.80	−3.60, 11.00	0.310
251–500		8.00	0.16, 16.00	**0.046**	4.90	−3.90, 14.00	0.277
>500		−6.30	−16.00, 3.80	0.222	−9.10	−20.00, 1.30	0.087
**Stroke Type**	187						
Ischemic Stroke		1.00			1.00		
Intracerebral Hemorrhagic Stroke		4.5	−3.8, 13	0.283	−0.37	−8.50, 7.8.00	0.928
Untyped Stroke		−32	−62, −1.1	**0.042**	−29.00	−58.00, −0.66	**0.045**
**Body Mass Index**	205	0.65	0.16, 1.10	**0.010**			
**Barthels Index**	214	0.23	0.14, 0.33	**<0.001**	0.22	0.12, 0.33	**<0.001**

1 CI = Confidence Interval.
